# Neuroimmunological Implications of AQP4 in Astrocytes

**DOI:** 10.3390/ijms17081306

**Published:** 2016-08-10

**Authors:** Hiroko Ikeshima-Kataoka

**Affiliations:** 1 Department of Pharmacology and Neuroscience, Keio University School of Medicine, 35 Shinanomachi, Shinjuku-ku, Tokyo 160-8582, Japan; ikeshima@1988.jukuin.keio.ac.jp; Tel.: +81-3-5363-3750; Fax: +81-3-3359-8889; 2 Faculty of Science and Engineering, Waseda University, 3-4-1 Okubo, Shinjuku-ku, Tokyo 169-8555, Japan

**Keywords:** astrocyte, aquaporin 4 (AQP4), blood-brain barrier (BBB), central nervous system (CNS), endofoot, glial fibrillary acidic protein (GFAP), gliosis, interleukin (IL)-1β, IL-6, immunoglobulin G (IgG), microglia, neuromyelitis optica (NMO), neuroimmunology, osteopontin (OPN), reactive astrocyte, tumor necrosis factor (TNF)-α

## Abstract

The brain has high-order functions and is composed of several kinds of cells, such as neurons and glial cells. It is becoming clear that many kinds of neurodegenerative diseases are more-or-less influenced by astrocytes, which are a type of glial cell. Aquaporin-4 (AQP4), a membrane-bound protein that regulates water permeability is a member of the aquaporin family of water channel proteins that is expressed in the endfeet of astrocytes in the central nervous system (CNS). Recently, AQP4 has been shown to function, not only as a water channel protein, but also as an adhesion molecule that is involved in cell migration and neuroexcitation, synaptic plasticity, and learning/memory through mechanisms involved in long-term potentiation or long-term depression. The most extensively examined role of AQP4 is its ability to act as a neuroimmunological inducer. Previously, we showed that AQP4 plays an important role in neuroimmunological functions in injured mouse brain in concert with the proinflammatory inducer osteopontin (OPN). The aim of this review is to summarize the functional implication of AQP4, focusing especially on its neuroimmunological roles. This review is a good opportunity to compile recent knowledge and could contribute to the therapeutic treatment of autoimmune diseases through strategies targeting AQP4. Finally, the author would like to hypothesize on AQP4’s role in interaction between reactive astrocytes and reactive microglial cells, which might occur in neurodegenerative diseases. Furthermore, a therapeutic strategy for AQP4-related neurodegenerative diseases is proposed.

## 1. Introduction

### 1.1. Aquaporin-4 (AQP4)

Aquaporin 4 (AQP4) is the most abundantly expressed water channel in the brain, and is highly localized in the endfeet of astrocytes (a type of glial cell in the central nervous system (CNS)); these endfeet are in contact with blood vessels [1,2,3]. The distribution of AQP4 is diverse throughout the brain, and includes the cerebral cortex, corpus callosum, retina, cerebellum, magnocellular nuclei of the hypothalamus, and brain stem [4]. AQP4 has a tetrametric structure, enabling gases and ions to permeate through a central pore; however, the physiological role of this central pore remains unclear [1]. Use of stopped-flow analysis showed that mercury decreases AQP4 M23 water permeability in proteoliposomes via Cys^178^ residue located cytoplasmic loop D [5]. AQP4 transfected astrocyte cell line and primary culture of astrocytes revealed that lead (Pb^2+^) increased water permeability, mediated by Ser^111^, which is a phosphorylation site for calmodulin kinase II (CaMKII) [6]. Oocytes expressing rat AQP4 exhibit greater permeability for CO_2_ but lower permeability for NH_3_ [7]. AQP4 could protect the brain from rising NH_3_ levels in the blood, while allowing CO_2_ to pass.

AQP4 is involved in astrocyte migration when water passes through the lamellipodium and into the cytoplasm by an osmotic gradient. Furthermore, AQP4 plays a role in neuroexcitation, in which isosmolar K^+^ is released by neurons, followed by the uptake of K^+^ and water by astrocytes on the other side of the synaptic cleft [3]. The inwardly rectifying K^+^ channel family member, Kir4.1, is co-localized with AQP4 at the endfeet of astrocytes, but not in neurons, to maintain water homeostasis in the CNS. These transmembrane channels seem to play important roles in neurological disorders [8].

The upregulation of metabotropic glutamate receptor (mGluR) 3 can be co-localized with AQP4, suggesting that astrocytic mGluR plays a role in the regulation of extracellular glutamate levels. In the hippocampal tissue of patients with temporal lobe epilepsy (TLE), the expressions of connexin 43 and AQP4 are increased, and the expressions of the key constituents of the AQP4 multi-molecular complex (Kir4.1, a-syntrophin, and dystrophin) are downregulated [9].

Hypotonicity induces a rapid and reversible relocalization of AQP4 in a calcium-, calmodulin-, and kinase-dependent manner in primary cortical rat astrocytes and transfected HEK293 cells [10].

AQP4 also reportedly plays a role in the development and maintenance of the blood-brain barrier (BBB) [11,12]; however, a detailed analysis, which included electron microscopy studies, revealed that the deletion of AQP4 does not alter BBB integrity or brain morphology [13]. We also examined the leakage of immunoglobulin G (IgG) to confirm BBB integrity in an independently established line of AQP4-deficient mice, compared with wild-type (WT) mice, and found that, not only was the integrity of the BBB maintained in a normal brain, but the recovery of the BBB after breakdown was also not altered, even in the absence of AQP4 [14].

AQP4 has also been implicated in learning and memory in the hippocampus and amygdala by influencing long-term potentiation (LTP) and long-term depression (LTD) [15]. Some evidence using AQP4-deficient mice has shown that LTP and LTD alterations are dependent on brain-derived neurotrophic factors [16], and a link between defective LTP and the downregulation of glutamate transporter-1 has been shown [17,18].

The deletion of AQP4 has been shown to result in the shrinkage of the extracellular space (ECS) volume in the mouse hippocampal CA1 region, which is associated with the activation of excitatory pathways [19]. ECS shrinkage was most pronounced in the pyramidal cell layer. These results imply that AQP4 regulates the dynamics of the extracellular volume.

### 1.2. Astrocytes

#### 1.2.1. Glial Fibrillary Acidic Protein

Astrocytes are a type of glial cell in the CNS that accounts for more than 50% of the total cells in the brain, and astrocytes are thought to be 10 times more abundant than neurons. Glial fibrillary acidic protein (GFAP) is an intermediate filament protein that is highly expressed in astrocytes and is considered to be an astrocyte marker [20]. The upregulation of GFAP can be observed in reactive astrocytes; such upregulation can be induced by traumatic injury, edema, inflammation, or infection in the brain. GFAP mutations cause Alexander disease, which is a fatal neurodegenerative disorder, characterized by astrocytic inclusions [21].

Several laboratories have independently developed GFAP-null mice, and such mice were found to develop normally, with no differences from WT mice in terms of brain architecture, numbers of neurons and astrocytes, BBB integrity, behavior, or motor activity (reviewed in [22]). At 14 months of age, however, GFAP-null mice develop hydrocephalus and reduced myelination in the corpus callosum, spinal cord, and optic nerve [23]. Thus, astrocytes might play a pivotal role in the long-term maintenance of myelination.

Another astrocyte marker, tenascin-C (TN-C), is an extracellular matrix molecule (ECM) that is expressed in radial glia in the CNS during development [24,25], and in primary cultures of astrocytes [26], while its expression is attenuated in astrocytes in the normal brain. In brains that have experienced traumatic injury, inflammation, or infection, the expression of TN-C is enhanced simultaneously with the expression of GFAP in reactive astrocytes [27,28]. Because of the inhibition of the activation of astrocytes and microglial cells in AQP4-deficient mice, TN-C expression was reduced in mouse brains with stab wound injuries or primary cultures of astrocytes stimulated with lipopolysaccharide (LPS). Thus, the drastic expression of TN-C in reactive astrocytes might depend on AQP4 expression [14].

#### 1.2.2. Oxidative Stress in Astrocytes

Oxidative stress is thought to be one of several causes of brain aging. Astrocytes contribute to neuronal homeostasis and couple with neurons in their response to oxidative stress so as to protect them [29]. Aging regulator insulin-like growth factor 1 (IGF-1) directly participates in astrocyte neuroprotection against oxidative stress [30]. Superfusion of retinal slices with a hypoosmolar solution induced a rapid swelling of Müller cell somata in tissues from AQP4-deficient mice, but not from wild-type mice [31]. AQP4 is involved in the rapid volume regulation of retinal glial cells in response to osmotic stress and that deletion of AQP4 results in an inflammatory response of the retinal tissue.

#### 1.2.3. Calcium Signaling in Astrocytes

Astrocytes rapidly swell during brain edema formation [32], and brain swelling triggers Ca^2+^ signaling in astrocytes; this signaling is reduced in AQP4-deficient mice. Thus, hypo-osmotic stress initiates astrocytic Ca^2+^ spikes in an AQP4-dependent manner [33]. The calcium dynamics in astrocytes have been thoroughly examined in both primary cultures and genetically modified awake mice, as previously reviewed [34,35,36].

#### 1.2.4. Astrocytes and Immune Cells

Astrocytes interact with T-cells in inflammatory responses via T-cell receptor (TCR) and adhesion molecule lymphocyte function-associated antigen 1 (LFA-1), the so-called T-cell-astrocyte interface, to form immunological synapses (ISs). Bacterial LPS or tumor necrosis factor (TNF-α) strongly stimulates astrocytes to release chemokines [37]. In this manner, astrocytes act as chemokine producers or lymphocyte attractants for the recruitment of T-cell subsets into the brain parenchyma [38]. Immune modulator mesencephalic astrocyte-derived neurotrophic factor (MANF) has been identified in immune cells, and this biases immune cells toward an anti-inflammatory phenotype, enhanced neuroprotection and tissue repair, and improved the success of photoreceptor replacement therapies in both mouse and Drosophila [39]. Astrocytes, as well as microglia and macrophages, were important sources of IL-27 in the human disease, multiple sclerosis (MS). IL-27 triggered the phosphorylation of the transcription regulator STAT1, and can modulate immune properties of astrocytes and infiltrating immune cells in an MS patient’s brain [40].

## 2. Neuroimmunological Role of AQP4

### 2.1. AQP4 in Neuromyelitis Optica (NMO)

Neuromyelitis optica (NMO) is an autoimmune disease consisting of recurrent optic neuritis and transverse myelitis, and serologic testing for the AQP4-immunoglobulin G (IgG) autoantibody is useful for a differential diagnosis from multiple sclerosis (MS) [41,42,43]. Astrocytes in the optic nerve and spinal cord are the main targets. The loss of AQP4 and GFAP staining in NMO brain is distinct from the staining observed in MS patients [44]. An enzyme-linked immunosorbent assay (ELISA) to detect anti-AQP4 antibodies has been established, and can be used as a substitute for the conventional NMO-IgG assay [45]. Rat astrocytes and oligodendrocytes from primary cultures and rat optic nerves were exposed for 24 h to neuromyelitis optica (NMO)-IgG in the absence of complement, and it was found that there was a complement-independent effect of NMO-IgG/AQP4 antibody on astrocytes, with secondary damage to oligodendrocytes, possibly resulting from glutamate-mediated excitotoxicity [46].

Recently, our colleagues also established high-affinity monoclonal antibodies against the extracellular domains of AQP4, and these antibodies can block the binding of NMO-IgG, despite its heterogeneity. These antibodies could be applied in clinical treatments for NMO patients [47,48,49].

Highly pathogenic AQP4-peptide-specific T cells in Lewis rats have been reported. These cells recognize the AQP_268–285_ epitope and produce NMO-like lesions in the presence of NMO-IgG [50]. Comprehensive mutagenesis of the three extracellular loops of the M23 isoform of human AQP4 were analyzed, and the effects on binding of NMO AQP4-reactive recombinant IgG (rAbs) using quantitative immunofluorescence were evaluated. Amino acid substitutions at T^137^/P^138^ altered loop C conformation and abolished the binding of all NMO rAbs and NMO-IgG, and the authors concluded on the importance of loop C conformation to the recognition of AQP4 by pathogenic NMO Abs [51].

Another AQP4-autoimmunity disease, NMO-spectrum disorder (NMOSD), classifies, not only optic neuritis and myelitis as NMO, but also cerebral, diencephalic, brainstem, and area postrema syndromes [52,53]. Furthermore, non-neurologic features involving other AQP4-positive organs outside of the CNS have also been reported for NMOSD. Nevertheless, NMOSD can be treated by B-cell depletion through antibodies, or the infusion of “aquaporumab” to block AQP4 antibodies [54,55].

Myasthenia gravis (MG) is a disease affecting the neuromuscular junction, caused in approximately 85% of patients by IgG1- and IgG3-complement activating antibodies against the nicotinic acetylcholine receptor (AChR-Ab) [56]. Several cases or small series of MG patients show both NMO/NMOSD. A history of thymectomy for MG patients could be a possible risk factor for the later development of NMOSD [57]. Antibody titers for AQP4-Abs and AChR-Abs tend to change in opposite directions [58].

### 2.2. AQP4 in Alzheimer’s Disease

A defect in the clearance of β-amyloid (Aβ) in brain parenchyma is considered to be a cause of Alzheimer disease (AD) [59]. Astrocytes play a protective role in the clearance and degradation of Aβ through the recruitment of astrocytes toward monocyte chemoattractant protein-1 (MCP-1) in senile plaques [60]. However, the excessive uptake of Aβ causes astrocyte malformation and apoptosis [61,62]. AQP4 deficiency in cultured astrocytes resulted in reduced astrocyte activation induced by Aβ_1–42_ and its toxicity, the uptake of Aβ_1–42_, and the upregulation of LRP-1 induced by Aβ_1–42_, as well as altered levels of MAPK phosphorylation [63]. Thus, AQP4 in astrocytes is a molecular target for the treatment of AD. Moreover, direct evidence of interactions between AQP4 and glutamate transporter-1 (GLT-1) has been reported in astrocytes using AQP4-deficient mice, and these two proteins are part of the same supramolecular complex [64]. The collaboration of AQP4 and GLT-1 in astrocytes has a protective effect against glutamate-induced neuronal injury by Aβ, which might play a pivotal role in the regulation of distinct cellular responses that involve neuroprotection against AD [65].

### 2.3. AQP4 in Parkinson’s Disease

Parkinson disease (PD) is clinically characterized by the progressive, selective, and irreversible loss of dopaminergic (DA) neurons in the substantia nigra (SN) resulting in a poverty of voluntary movements (akinesia), slowness and impaired voluntary movement (bradykinesia), muscle rigidity, and tremors of the limbs at rest [66]. The administration of MPTP (1-methyl-4-phenyl-1,2,3,6-tetrahydropyridine)/probenecid in a PD mouse model with an AQP4 deficiency resulted in significantly enhanced gliosis, the aggravated loss of TH-immunoreactive neurons, an increase in the production of IL-1β and TNF-α, but a suppression of IL-6 in the midbrain, and the activation of the IKK/NF-κB pathway in vivo, compared with WT mice [67]. Neurotoxicity induced by AQP4 was observed, not only in the SN, but also in ventral tegmental area (VTA) neurons [68]. Another paper also reported that AQP-deficient mice showed hypersensitive to stimulation of MPTP or LPS compared to WT littermates [69]. MPTP-induced PD mouse model with AQP4-deficient mice showed more robust microglial inflammatory responses and more severe loss of DA neurons. Significantly lower numbers of CD4^+^ CD25^+^ regulatory T cells in AQP4-deficient mice compared to WT. The authors reported for the first time for AQP4 expression in mouse thymus, spleen, and lymph nodes. Thus, they concluded that AQP4 may have immunosuppressive regulator function.

### 2.4. AQP4 in Depression

AQP4 may serve as a marker for an astrocytic pathology in major depressive disorder [70,71]. Mesenchymal stem cell therapy can repress inflammation during ischemic stroke in mice, thereby protecting the integrity of the blood-brain barrier, reducing brain edema and astrocyte apoptosis, and downregulating AQP4 expression via the p38 signaling pathway [72]. AQP4-deficient mice showed an improved neurological outcome after acute water intoxication and ischemic stroke [73]. In human glioma cells, AQP4 regulates migration and invasion and might be useful as a therapeutic target for cell infiltration [74].

### 2.5. AQP4 in Blood-Retinal Barrier Breakdown

Blood-retinal barrier (BRB) breakdown occurs in diabetic retinopathy, age-related macular degeneration, retinal vein occlusions, and uveitis, resulting in vasogenic edema and a loss of vision because of neural tissue damage [75]. AQP4 deletion is directly responsible for BRB dysfunction to the deep plexus capillaries, and strong GFAP upregulation was observed in astrocytes in the retina, while the expression of glutamate synthetase (GS), a Müller cell marker, was not observed [76], even though AQP4 was expressed in both types of cells. Since AQP4 expression is not homogeneous among all astrocytes, neither in mouse brains or primary cultures [14], Müller cell dysfunction might also be not homogeneous.

An interaction between transient receptor potential isoform 4 (TRPV4) and AQP4 has been proposed as a key regulator in astroglial swelling, volume regulation, and the reorganization of downstream signaling pathways in retinal Müller cells [77], and the coordination of activity-dependent ionic/water fluxes at the BRB might be critically dependent on functional interactions among TRPV4, AQP4, and Kir4.1 channels.

### 2.6. AQP4 in Traumatic Brain Injury

Traumatic brain injury causes brain edema resulting from an increased brain volume as a result of water uptake, with elevated intracranial pressures leading to brain herniation and neuronal death [78,79]. Vasopressin 1a receptor (V1aR) antagonists prevent brain edema, rescue astrocytic cell swelling, and attenuate GFAP and AQP4 expression after cortical contusion injury [80]. In short, V1aR inhibitors might be useful tools for reducing brain edema in future clinical studies.

AQP4 is known to contribute to cytotoxic edema following traumatic brain injury (TBI). TBI leads to the transcriptional activation of Foxo3a, a mammalian forkhead transcriptional factor, and the upregulation of AQP4 in astrocytes at the injured site at 24 h after TBI [81]. Foxo3a directly binds to the AQP4 promoter (binding residue, ATAAACA), as verified using a gel shift assay and a chromatin immunoprecipitation assay. Furthermore, the depletion of Foxo3a reduces the induction of AQP4 and cerebral edema in TBI mice.

A brain-wide network of paravascular channels, termed the “glymphatic” pathway, exists. In this pathway, subarachnoid cerebrospinal fluid (CSF) recirculates through the brain parenchyma along the paravascular spaces, exchanging with the surrounding interstitial fluid (ISF) to facilitate the clearance of interstitial solutes [82,83]. The paravascular CSF-ISF exchange and interstitial solute clearance depends on AQP4. TBI causes the loss of perivascular AQP4 polarization in the astrocytic endfeet in AQP4-deficient mice, impairing the paravascular clearance of interstitial solutes such as Aβ [84]. Furthermore, AQP4-deficiency promotes neurodegeneration and neuroinflammation, thereby exacerbating post-traumatic tau aggregation and cognitive impairment [82].

We have been analyzing a stab wound injury mouse model to clarify the functional role of astrocyte activation after brain injury. Using a microarray analysis, we found that AQP4 has neuroimmunological functions in injured mouse brain that are correlated with the pro-inflammatory cytokine inducer osteopontin (OPN) [85].

OPN plays numerous roles in immune-related diseases such as multiple sclerosis (MS), rheumatoid arthritis, lupus-related diseases, Sjögren syndrome, and colitis, and it also plays an important protective role in the immune response [86] and might contribute to MS lesions and NMO pathology because of the elevated production of OPN in cerebrospinal fluid [87,88].

### 2.7. AQP4 in Ischemia

In a normal CNS, AQP4 is only expressed in the endfeet of astrocytes, but its expression is dispersed throughout the cytoplasm of activated astrocytes. In bilateral carotid artery occlusion (BCAO), AQP4 deletion caused a reduction in astrocyte swelling and water accumulation in the brain, resulting in reduced BBB disruption, inflammation, and neuronal death [89]. Furthermore, AQP4 deficiency in mice improved the neurological outcome and exerted a neuroprotective effect against severe global cerebral ischemia [90].

Mitogen-activated protein kinase (MAPK) signal pathways are involved in changes in osmolality, and these pathways mediate AQP4 expression in an ischemia model, such as oxygen-glucose deprivation (OGD) in rat cortical astrocytes and middle cerebral artery occlusion (MCAO) in rats [91]. A p38 inhibitor protected against astrocyte cell death after OGD and restored AQP4 expression, attenuating edema and the infarct volumes after MCAO.

## 3. AQP4 in Reactive Astrocytes

When the brain experiences a traumatic injury or inflammation, astrocytes become active and engage in proliferation, migration, and the upregulation of some marker proteins as well as the enhanced production of pro-inflammatory cytokines and the formation of a glial (consisting of reactive astrocytes and reactive microglia) scar [92,93]. AQP4 localizes, not only in endofeet, but is also dispersed in cytoplasm of reactive astrocytes [1].

Methylmercury (MeHg)-treated common marmosets showed lesions in the cerebrum, cerebellum, and peripheral nerves as a model of Minamata disease [94,95], and a slight increase in AQP4 was observed in the reactive astrocytes [96].

To understand the reactive astrocyte state, a microarray analysis was performed using two mouse injury models: ischemic stroke and LPS-induced neuroinflammation [97]. The analysis revealed that reactive astrocytes induced during ischemia might be neuroprotective, whereas reactive astrocytes induced by LPS may be detrimental. The authors concluded that reactive astrocytes are highly heterogeneous, although AQP4 expression is observed in both types of reactive astrocytes. Our previous observations of primary culture of astrocytes showed that AQP4 expression is not homogeneous in either the brain or primary cultures [14], suggesting that astrocytes could be classified into multiple groups according to their AQP4 expression levels.

GFAP expression was intensively upregulated in reactive astrocytes at three days after a stab wound injury to the cerebral cortex, with the same kinetics observed for TN-C and AQP4 [14]. We found that the robust expressions of GFAP and TN-C were attenuated in stab wound injuries of the brain or in LPS-treated primary culture of astrocytes from AQP4-deficient mice, suggesting that the expressions of GFAP and TN-C in reactive astrocytes are dependent on AQP4 expression.

## 4. AQP4 in Neural Stem Cells

Reactive astrocytes and neural stem cells share many characteristic hallmarks, and neural stem or progenitor cells can reportedly be instructed to exhibit multipotency and long-term self-renewal upon exposure to growth factors in vitro [98,99]. Interestingly, AQP4 is expressed in the adult forebrain subventricular zone (SVZ), where neural stem cells (NSCs) are known to reside [100]. The isolation and culture propagation of adult murine and human SVZ-derived NSCs (ANSCs) are differentially regulated at different stages of differentiation and maturation into neurons and glia, either astrocytes or oligodendrocytes. GFAP is also expressed in both NSCs and ANSCs with the potential to differentiate into neurons and glial cells [101]. The parallel expression of GFAP and AQP4 might be a good marker, not only for differentiated astrocytes or reactive astrocytes, but also for precursor cells. Unfortunately, AQP4 expression in induced pluripotent stem cells (iPS) has not yet been reported.

## 5. AQP4 in Microglia

Microglia are the resident macrophages in the CNS, and they can dramatically transform from a resting state, such as a “surveying ramified state”, to an active state, such as an “amoeboid state” [102]. Microglial cells can hurriedly communicate, as if they are surveying, with astrocytes, oligodendrocytes, and neurons not only while they are in an active state, but also while they are at resting state [103,104]. Thus, microglia can promptly respond to damage signals in the brain within minutes, producing pro-inflammatory cytokines to protect neuronal cells from secondary damage [105]. Since astrocytes express receptors against interleukin (IL)-1β, IL-6, and tumor necrosis factor (TNF)-α the pro-inflammatory cytokines released from microglia may lead to the activation of astrocytes [106,107].

The intranigral injection of lipopolysaccharide (LPS) to induce neurotoxicity causes the activation of microglia, the loss of reactive astrocytes, the disruption of the BBB, and vasogenic edema in rats [108]. Activated microglial cells in the substantia nigra (SN) express AQP4 mRNA and protein in response to LPS injection. We also observed that AQP4 is expressed in activated microglial cells induced by a stab wound brain injury in mice [85]. However, the intracerebral injection of LPS did not induce AQP4 expression in reactive microglia [109]. These differences might depend on the procedure used to stimulate microglial activation or the microglial residence in the brain, but further studies are needed.

## 6. AQP4 Function in Astrocyte and Microglial Communication

Astrocyte and microglia interactions are required for LPS to induce the expression of pro-inflammatory cytokines and glial cell line-derived neurotrophic factor (GDNF) in astrocytes. Furthermore, microglia-derived TNF-α plays a pivotal role as a paracrine signal for the neuroprotective functions of astrogliosis [110].

Astrocyte activation is promoted by reactive microglial cells in several neurodegenerative diseases, such as experimental autoimmune encephalomyelitis (EAE) and Alzheimer disease (AD), and microglial cells are activated earlier than astrocytes [111]. In human glioblastoma, orthogonal arrays of particles (OAPs) are redistributed to membrane domains because of the degradation of the proteoglycan agrin by the increased activity of matrix metalloprotease 3 (MMP3). Agrin binds with AQP4 and leads to a strong immunoreactivity distribution in tumor tissues such as astrocytomas or glioblastomas, so that it may facilitate infiltration into the brain parenchyma, whereas it is restricted to the perivascular endfeet in normal brain [112,113,114].

In a PD mouse model using AQP4-deficient mice, AQP4 deficiency promoted the activation of microglial cells when co-cultured with astrocytes and induced the release of ATP from astrocytes [67]. Thus, AQP4 might modulate astrocyte-to-microglia communication during neuroinflammation.

AQP4 expression is upregulated by high-mobility group box 1 (HMBG1) via microglia-astrocyte interactions. The intracerebroventricular (i.c.v.) injection of HMGB1 significantly increased AQP4 protein and induced edema in the brain [115]. Furthermore, they used a primary culture of astrocytes and microglia and found that through diffusible factors, such as IL-1β from microglia, HMGB1 indirectly upregulated AQP4 and translocated NF-κB to the nucleus in astrocytes.

**Figure 1:**
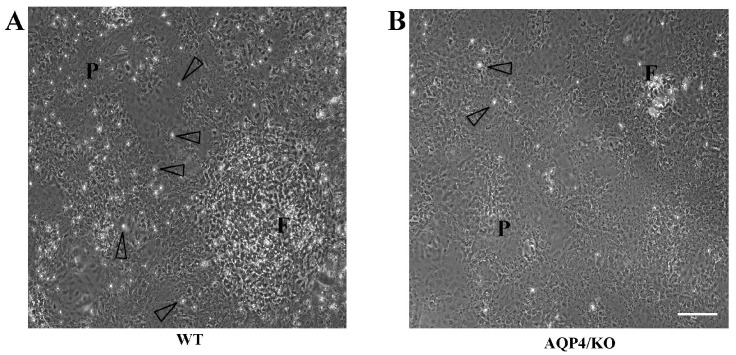
Primary cultures of astrocytes from WT mice are composed of tile-shaped cells known as protoplasmic astrocytes (designated as P in (**A**)) and rocky-shaped cells known as fibrous astrocytes (designated as F in (**A**)). On the other hand, most of the cells from the AQP4-deficient mice were protoplasmic astrocytes (**B**). Microglial cells are indicated by open arrowhead. Bar scale = 100 μm. Reproduced and modified from Reference [14], with permission Copyright © 2014 Wiley Periodicals, Inc.

Microglia and astrocytes are known to respond to cytokine challenges; however, a microarray analysis of human brain pericytes also revealed widespread changes in gene expression in response to interleukins and chemokines, and these changes might also be involved in BBB disruption [116]. The authors confirmed the translocation of nuclear factor NF-κB from the cytoplasm to the nuclei using primary human brain pericytes in the absence of microglia or astrocytes in responses to TNF-α, IL-1β, and LPS. The lack of astrocytic laminin induces the prevention of pericytes differentiation, inhibits AQP4 expression, and causes BBB breakdown in conditional knockout mice [117]. Brain pericytes surround endothelial cells and are in direct contact with astrocyte endfeet; thus, we must keep in mind that brain pericytes are responsible for neuroinflammation [118,119,120]. Pericytes could be another functional cell involved in neuroimmunological mechanisms. We recently reported that microglial cells attached to the primary culture of astrocytes from WT mice, as shown by the open arrowhead in Figure 1, but could not adhere to the astrocytes from AQP4-deficient mice. AQP4-mediated cell signals between astrocytes and microglia might be needed for the primary cultures. The atypical adhesion of microglia to astrocytes might be caused by a reduction in OPN or its receptors in primary culture of astrocytes and/or microglial cells because of the AQP4-deletion.

**Figure 2:**
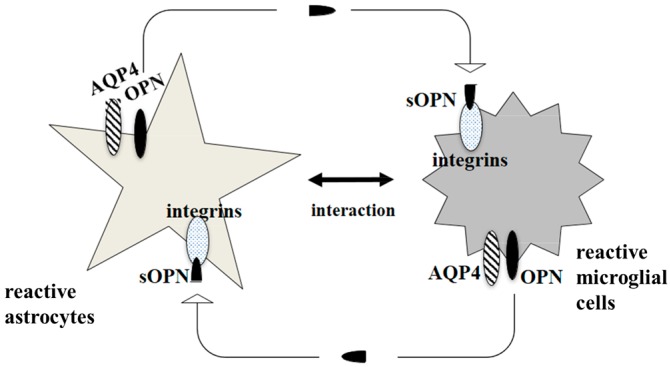
Involvement of AQP4 in communication between reactive astrocytes and reactive microglial cells. Direct interaction between AQP4 and intracellular form of osteopontin (iOPN) could be hypothesized, although has yet to be demonstrated. Alternatively, one of the integrin receptors for secreted form of osteopontin (sOPN) might enable an indirect interaction with AQP4.

The multiple functions of OPN are supported by its two isoforms, which are cleaved by thrombin or matrix metalloprotease: a secreted form of OPN (sOPN) and an intracellular form of OPN (iOPN) [121]. Receptors for sOPN include various integrin family members. We would like to hypothesise that AQP4 might provide a cooperative function in a neuroimmunological role with iOPN. Alternatively, one of the integrin receptors for sOPN might enable an indirect interaction with AQP4. A comprehensive analysis of AQP4 and OPN together might shed light on the treatment of autoimmune diseases in the CNS (Figure 2).

## 7. Conclusions

Because of the various functions of AQP4 in the brain, it might be useful to focus on AQP4 as a therapeutic target for neurodegenerative diseases. Recently, we found that injured brain induces expression of many kinds of genes involved in inflammation or immunological function, and were significantly attenuated in AQP4-deficient mice (underlined in Table 1). The list must be a useful information to give some hints that several molecules in it could be a target for the neurodegenerative diseases, such as complement-dependent cytotoxicity therapy, chimeric antigen receptor-T-cell therapy, or developing neutralizing antibody.

## Figures and Tables

**Table 1:** Top 20 upregulated genes analyzed in a microarray experiment comparison between without stab wound and 3 days after a stab wound to the cerebral cortex in WT and AQP4/KO mice categorized with gene function. Genes concerned in inflammation or immunological role are underlined. Values indicate fold change of expression level compared with without stab wound mice (D0). Reproduced and modified from Reference [85], with permission Copyright © 2013 Elsevier Limited, Inc.

Function	Gene Description	WT	AQP4/KO
immune response	secreted phosphoprotein 1 (Spp1) = osteopontin (OPN)	59.83	4.94
	lipocalin 2 (Lcn2)	11.79	<1.5
	macrophage expressed gene 1 (Mpeg1)	8.89	<1.5
	chitinase 3-like 1 (Chi3l1)	4.67	<1.5
	leukocyte immunoglobulin-like receptor, subfamily B, member 4 (Lilrb4)	4.36	<1.5
enzyme	heme oxygenase (decycling) 1 (Hmox1)	23.19	11.49
	transglutaminase 1, K polypeptide (Tgm1)	5.29	<1.5
lysosomal function	lysozyme 2 (Lyz2)	18.53	1.9
compliment activation	complement component 3a receptor 1 (C3ar1)	8.77	<1.5
	complement component 1, q subcomponent, C chain (C1qc)	6.14	<1.5
	complement component 1, q subcomponent, beta polypeptide (C1qb)	5.31	<1.5
cytoskeleton	vimentin (Vim)	7.03	<1.5
	glial fibrillary acidic protein (Gfap)	4.69	<1.5
	lymphocyte cytosolic protein 1 (Lcp1)	4.52	<1.5
antigen expression	CD180 antigen (Cd180)	6.38	<1.5
	CD68 antigen (Cd68)	6.21	2.96
	lymphocyte antigen 86 (Ly86)	5.19	<1.5
adipose function	adipose differentiation related protein (Adfp)	6.85	2.49
chemokine	chemokine (C-C motif) ligand 3 (Ccl3)	5.37	<1.5
signal transduction	membrane-spanning 4-domains, subfamily A, member 6C (Ms4a6c)	5.32	<1.5
